# Mr. Martin, on Cold Affusion in Fever

**Published:** 1808-07

**Authors:** P. J. Martin


### *To the Editors of the Medical and Physical Journal.*

GENTLEMEN,

THE following case is copied from a late publication called "Struggles through Life, or the Adventures of Lieutenant John Harriott, formerly of Rochford in Essex, now resident magistrate of the Thames Police; and as we have no reason to doubt the truth of this gentleman's narrative, I think it worthy of a place in your Journal. I have chosen to give it in his own words, as most of your readers, probably, will not have the book at hand to refer to.

Although the propriety of Mr. Harriott's practice, particularly with regard to the cold affusion, now wants no confirmation; it is a striking example, and shews how often we are anticipated, and how often we are outheroded, by amateurs and medical dabblers.

As Mr. H's narrative is so extremely defective in dates, I cannot say precisely when the circumstances here related happened, (as occurring at Bencoolen) but guess, from collateral information, that it must have been soon after the year 1770.

Can any of your readers give us an account of a treatise on the use of water, under the title of "Febrifugum Magnum," by John Hancock, D. D. Rector of St. Margaret's, Lothbury, London, and Prebendary of Canterbury, &c.? and also of "The Curiosities of Common Water," by John Smith, C. M. published 1740, soon after the former?—Perhaps they are well known in the medical world.

P. J. MARTIN.

---

"One morning, finding myself exceedingly out of order, two or three hours earlier than my usual time of rising, I was soon sensible, from the excessive burning heat all over me, that one of the same destructive fevers had seized me, which had so rapidly carried off many of the inhabitants. I had noticed the ill success of their medical treatment, and had resolved, in case of an attack, to be my own doctor as long as it was in my power; and I had directed my servant, who came with me from Madras, accordingly.

"I consulted my own sensations as to what I felt desirous of being done; and my predominant wish was, that I could roll myself in snow, as I had seen the American Indians. My head in particular was like a ball of fire; and I apprehended approaching distraction, so as to disable me from giving sound directions. Calling my servant, therefore, who lay in an adjoining passage, I ordered him to throw a handful or two of saltpetre into three earthen jars of water, and stir it as was usual for cooling our wine. As soon as this was ready, I went out, and made him pour the liquor from the three jars successively over my head. I felt instant relief; and wrapping a quilted soosee morning gown about me, lay down again, directing my servant to cover me with other clothes, in the hope of getting into a perspiration, but without effect.

"My stomach and bowels now seemed much oppressed, as if wanting relief. I was always provided with ipecacuanha, jalap, rhubarb, and salts, made up in doses: directing my servant to take two of the former and mix ready for taking, I swallowed this double dose, and, while I laid down again, ordered a tub to be brought to the side of my cot, and some very weak tamarind tea to be prepared. I suffered much before it took the least effect; at length it came with violence, nearly approaching to suffocation; nor was it long after, before it likewise operated downwards. Strong as I deemed myself, I found my strength exhausting very fast, so that I could scarcely turn myself quick enough for the alternate motions, and was at last obliged to order Peter to bring in another tub. I sat over one, and retched over the second, supported by my camp-cot on one side and my servant on the other. How long I kept the two pumps at work I cannot say, but it appeared a terrible long time. When the retching ceased I drank a little mulled wine and water, well cinnamoned, but had not sufficient strength left to rise and get into my cot without help.

"Weak

"Weak and exhausted as I was when I laid down, I felt comparative ease; and, after a while, taking a little more mulled wine, I sunk gradually into a sound sleep.

"To make short of my story, I recovered speedily, in defiance of Doctors M'C—— and M——, who, as soon as the circumstances were known from my servant, while I continued sleeping, declared I had killed myself beyond all power of redemption."

After apologizing for what he calls the rashness of his procedure, and his want of confidence in the regular practitioners, he goes on—

"For several years I had been in the constant habit of having large Cudjaree-pots of water thrown over me in a morning; and hundreds of times, during the violence of the hot land winds on the coast of Coromandel, when retired from company after dinner, finding it impossible to repose on account of the heat, I have seated myself on a camp-stool, in the most likely situation to feel an air of wind in the shade; and with nothing on but a banyan shirt and long drawers, have placed a towel soaked in water upon my head, keeping a second in a pot of cool water close to my side, ready to place on my head as the former one ceased draining; and in this situation have I continued for an hour or two, comfortably reading a book."

